# Supplementary material for: A memory transcriptome time course reveals essential long-term memory transcription factors
Source: Nat Commun. 2025 Oct 29;16:9320. doi: 10.1038/s41467-025-64379-x (PMC12572301; doi:10.1038/s41467-025-64379-x)
Supplement: Supplementary file 1 — Supplementary Information [file 41467_2025_64379_MOESM1_ESM.pdf]

Supplementary Figures

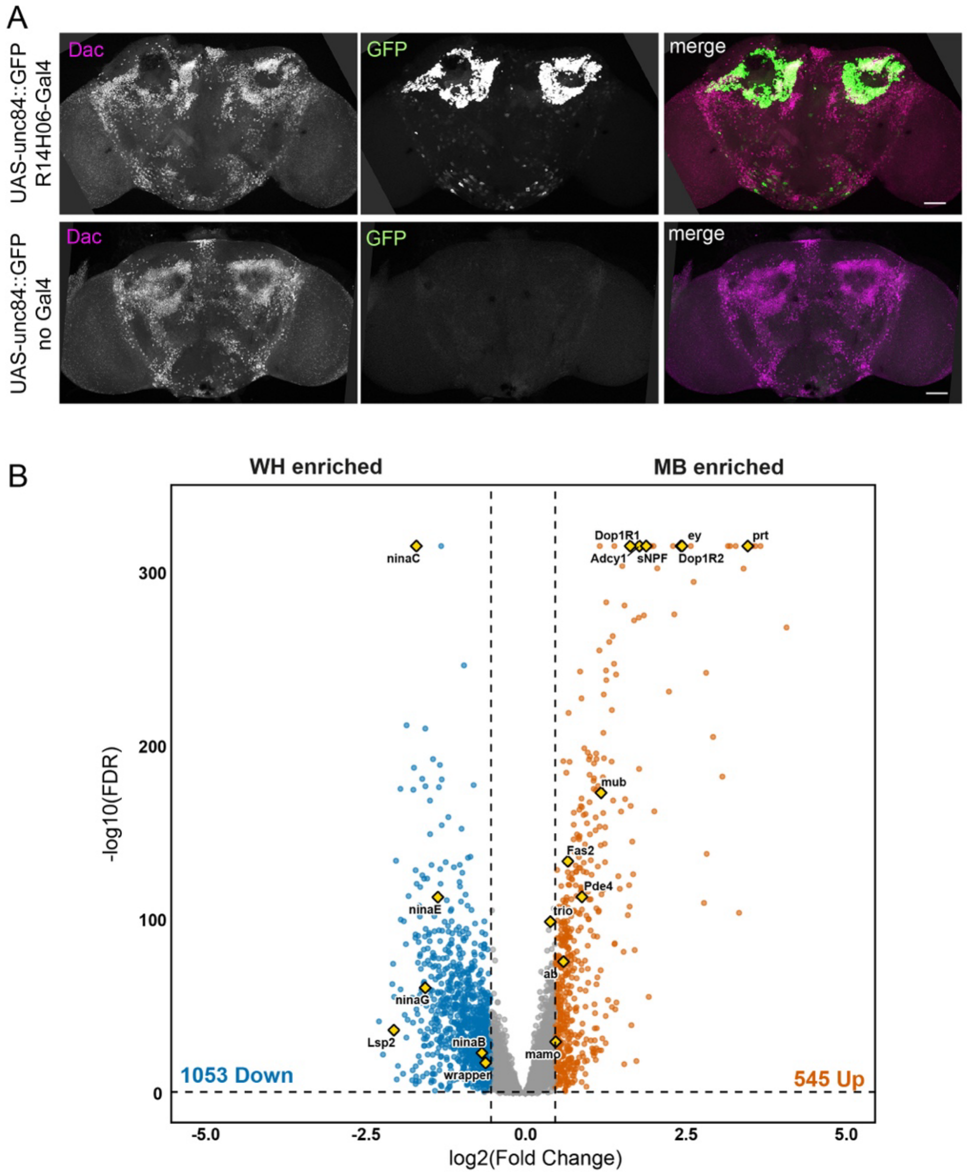

Figure S1 - INTACT yields mRNA enriched from MB nuclei. (A) Whole brain confocal projections showing specific expression of *UAS-unc84::GFP* in Kenyon cells (green). Kenyon cells are co-labeled with an anti-Dac antibody (magenta). While several non-MB nuclei are visible, most expression is within KCs. Scale bar represents 50μm (B) Volcano plot showing differentially expressed genes (DESeq2, Wald's test, FDR < 0.05) between whole-head and INTACT-purified mushroom-body (MB) nuclei. MB-enriched genes are highlighted in orange and WH-enriched genes are highlighted in blue. Notable MB and non-MB genes are indicated.

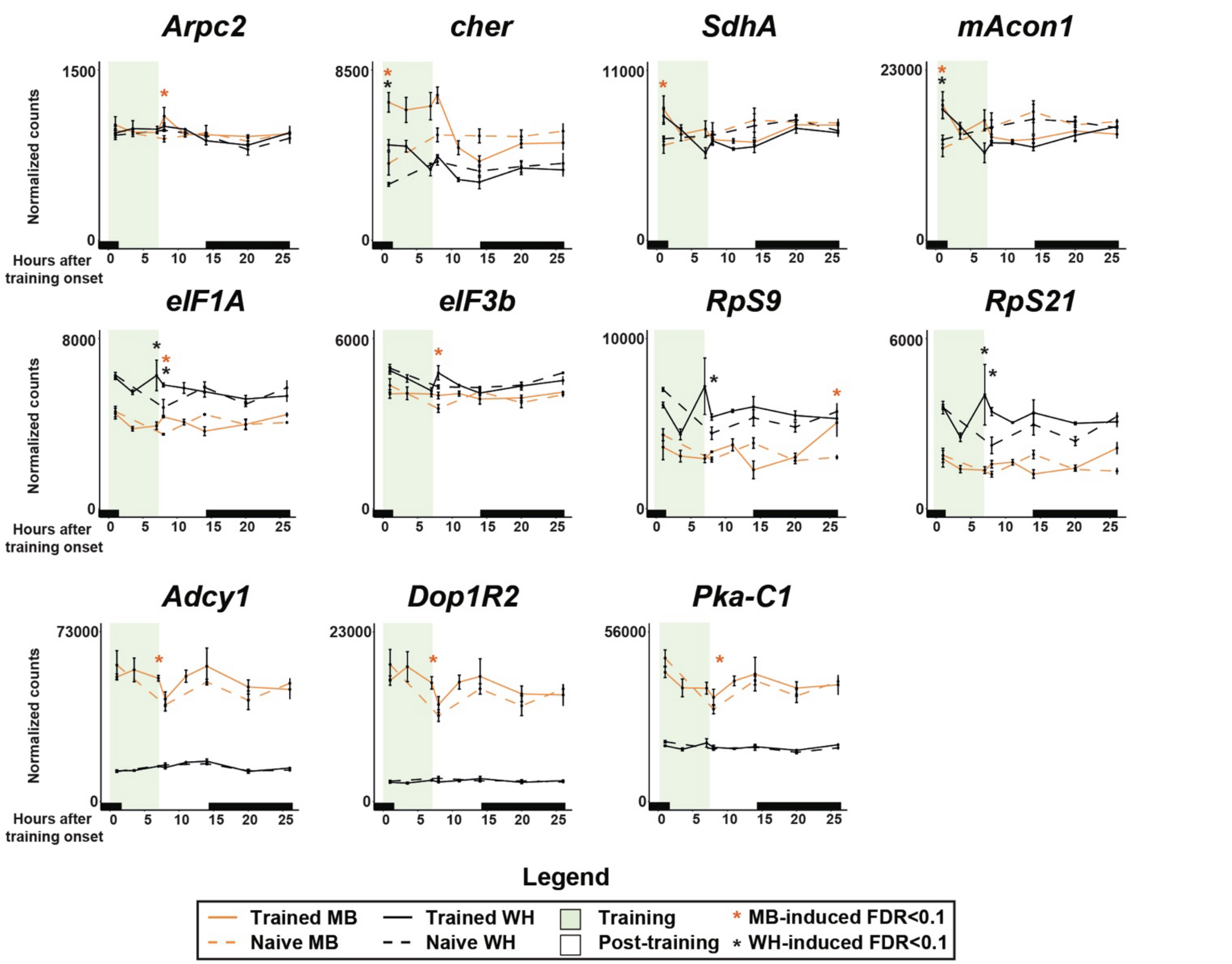

Figure S2 – Examples of individual training induced genes. Graphs showing average z-score normalized mRNA expression levels in MB and WH nuclei of naïve and trained flies, across the entire courtship memory time course. Gene names are indicated. Light and dark periods are indicated by white and black bars, respectively. Statistical analysis was done using Wald's test in DEseq2. Error bars represent standard error of the mean.

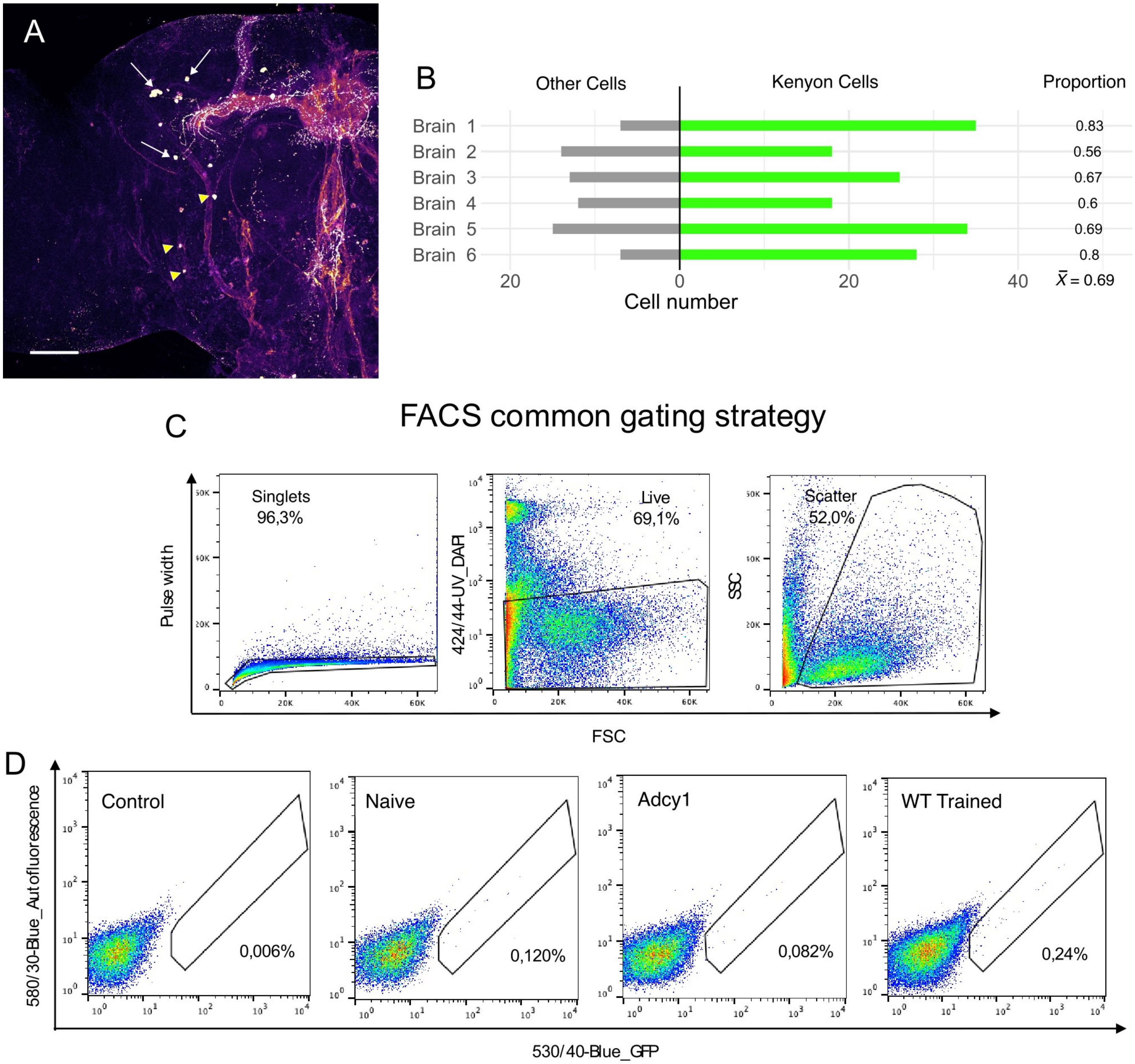

Figure S3 – CAMEL-GFP cell sorting of dissociated *Drosophila* brains. (A) Example of a hemi-brain depicting CAMEL-GFP positive Kenyon Cells (KCs, marked by arrows) and non-MB neurons (marked by triangles). Fascicline II staining (magenta) identifies the MB. Scale bar represents 50μm. (B) Relative quantification of KC CAMEL-GFP neurons in 6 *wt* brains. Source data are provided as a Source Data file. (C) The common gating strategy followed up on the sort to exclude cell aggregates, dead cells and cellular debris. (D) Percentage of the GFP positive events for control, WT naive, *Adcy1* mutant and WT trained conditions. GFP percentage refers to those single live non debris events.

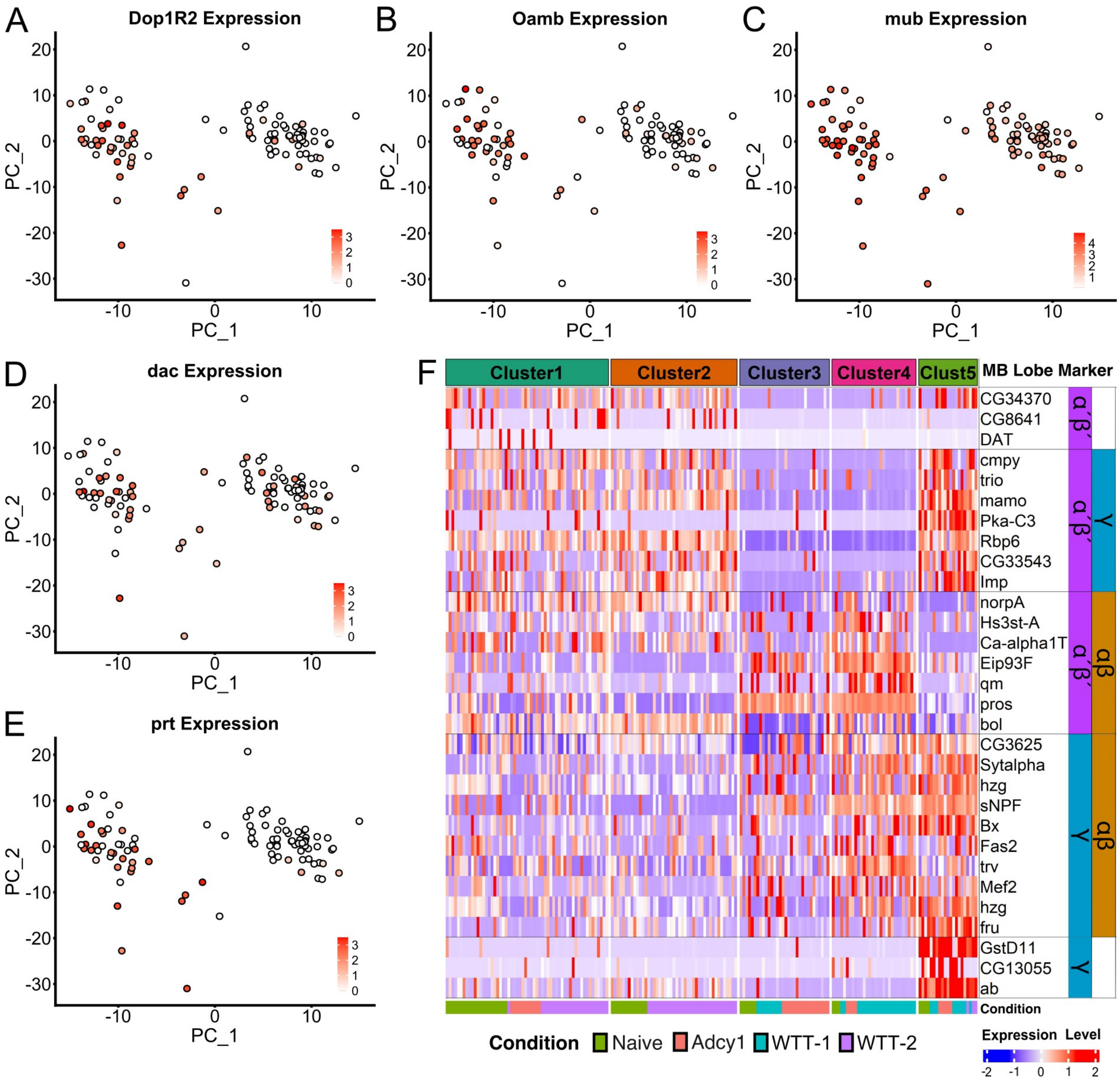

Figure S4 – Cellular identity of scRNAseq data. (A-E) PCA of WT trained flies depicting the expression (red) of general MB markers, such as *Dop1R2* (A), O*amb* (B), *mub* (C), *dac* (D) and *prt* (E). (F) Heatmap of 30 MB lobe markers. The lobe identity for markers is shown in lateral bars (αβ in brown, α´β´ in purple and γ in blue). Overlapping means that the marker is expressed in both lobes, although at different expression levels.

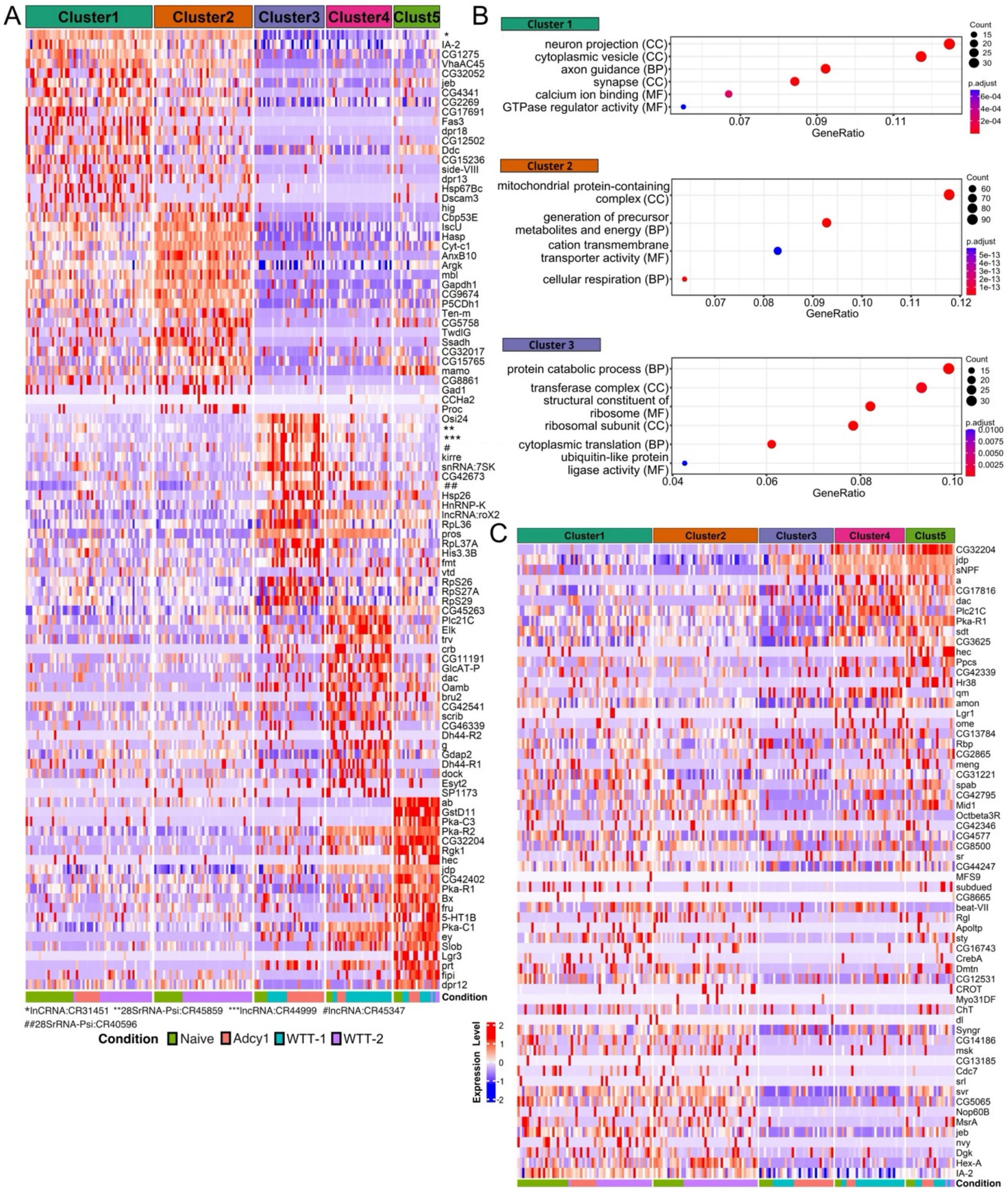

Figure S5 - Analysis of cluster 1-5. (A) Heatmap of top 20 most differentiating expressed genes between cluster 1-5. (B) Most representative GO terms (FDR <0.05) for clusters 1, 2 and 3, indicating cell component (CC), biological processes (BP) and molecular function (MF). (C) Heatmap of up-regulated genes after learning obtained by INTACT RNAseq following courtship conditioning. Genes are ordered by their expression levels in clusters 4 and 5.

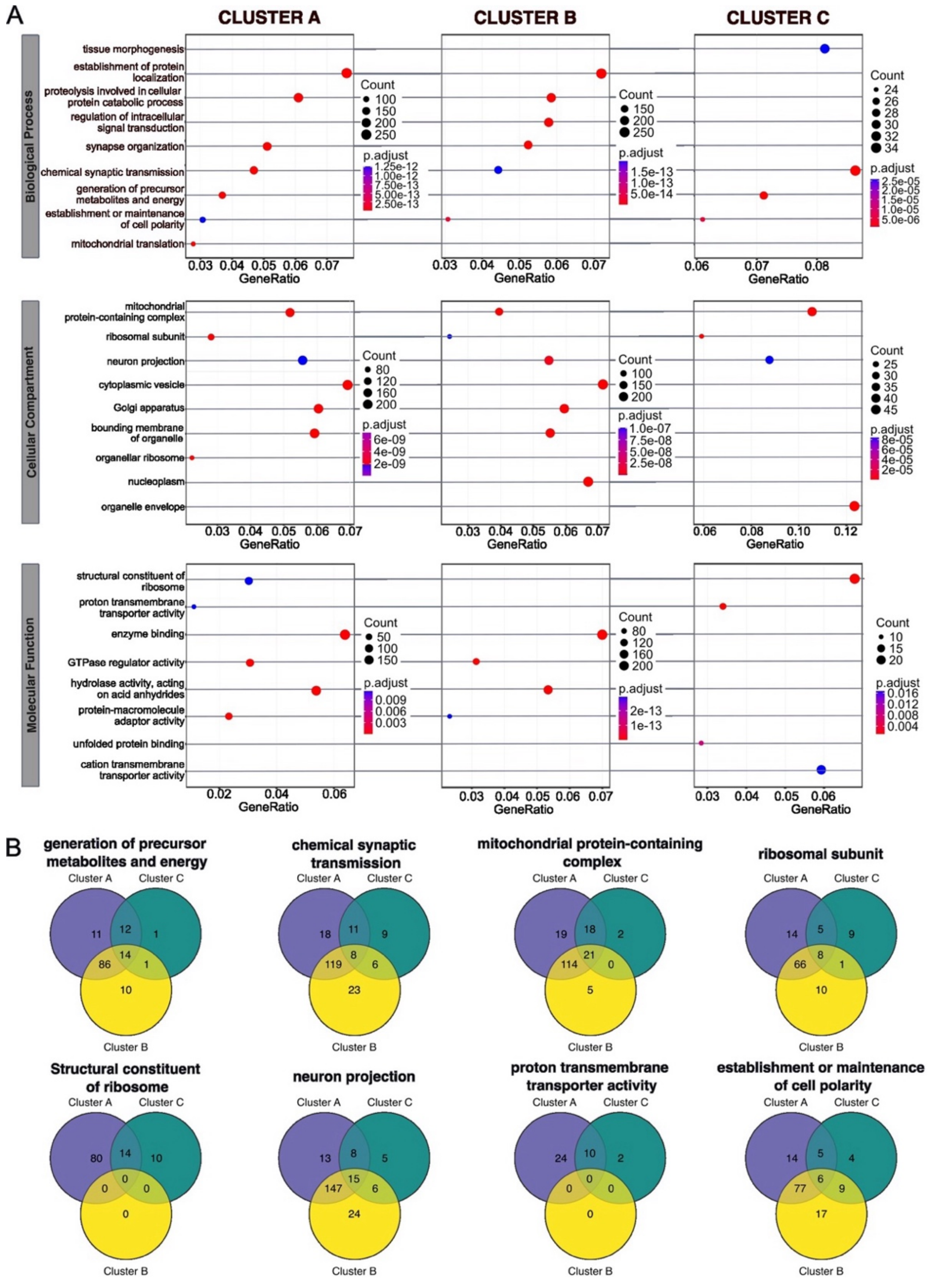

Figure S6 - Similar GO terms between cluster A-C. (A) Most representative of significative GO terms (FDR <0.05) between cluster A, B and C, categorized in biological processes, cellular component and molecular function. (B) Venn Diagram showing the number of genes for selected common GO terms between the three clusters (see also Suppl table 7).

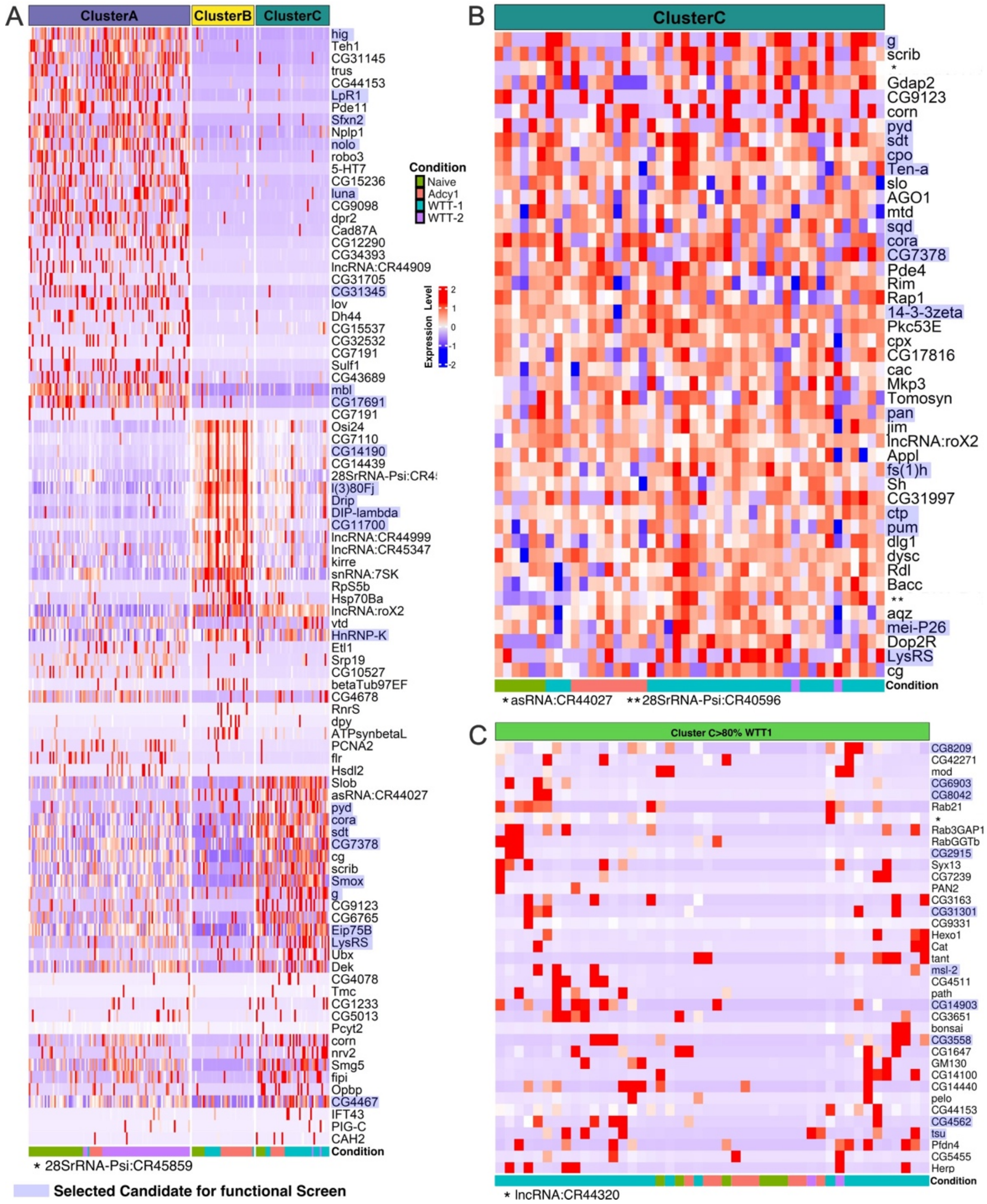

Figure S7- Selection of candidate genes. (A) Heatmap of top 30 most differentiating genes between cluster A, B, and C. (B) Heatmaps of cluster C genes most expressed in more than 50% of cluster C cells. (C) Heatmaps of cluster C>80% neurons. Selected candidate genes are cyan shaded (see Table S8 for details).

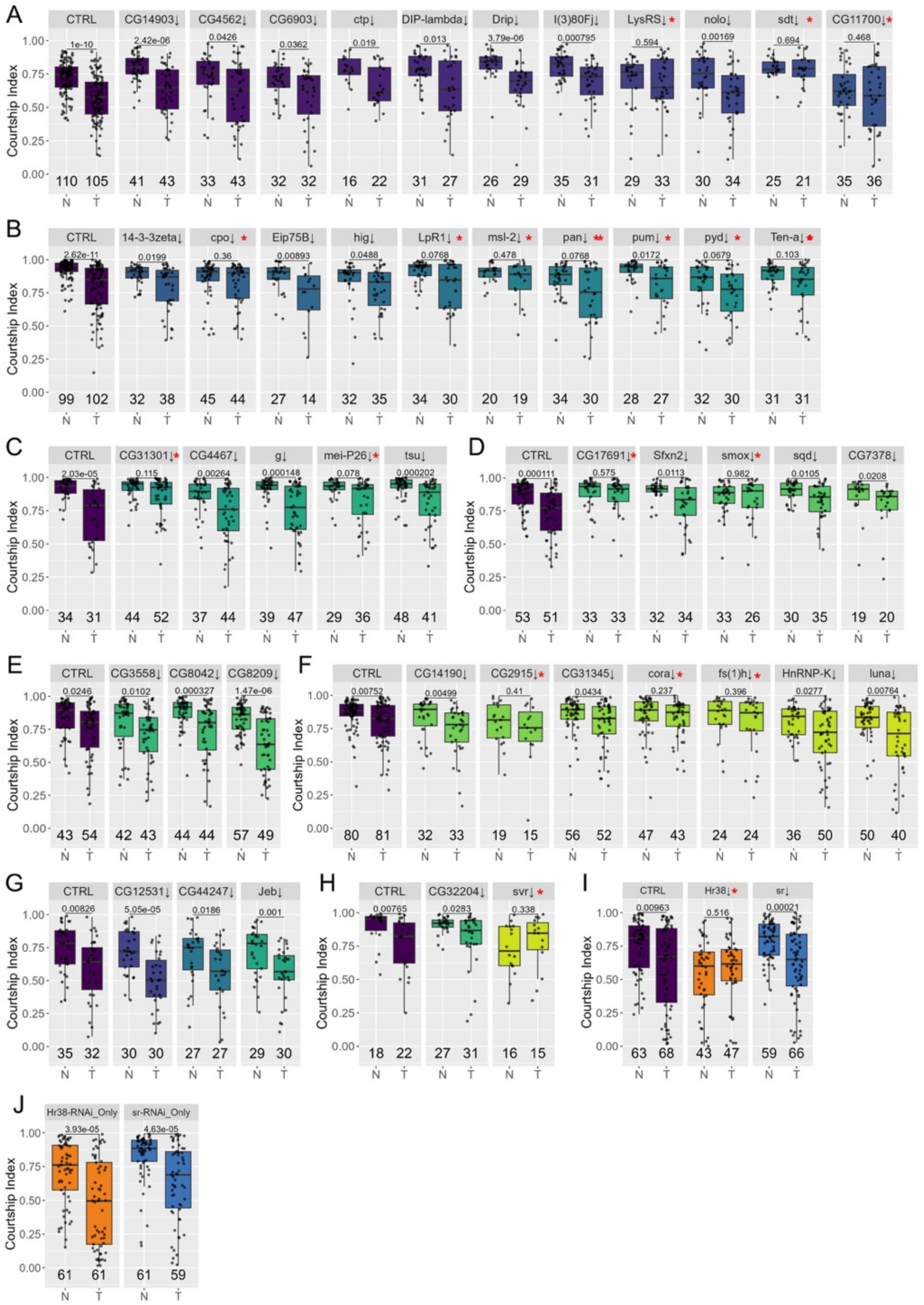

Figure S8 – Functional screen for genes governing Courtship LTM. (A-H) Box plots showing courtship indices (CIs) for naive (N) and trained (T) flies of 48 candidate genes tested

for courtship LTM. Each condition was repeated between 2-4 times. Red asterisks showed the positive hits that abolished LTM. (A) *MB247-Gal4* was crossed to short hairpin *UAS-RNAi* lines (Vallium20) from the TRiP collection in the AtttP2 landing site. The AttP2 genetic background strain (BDSC36303) was crossed to *MB247-Gal4* as a control. (B) *UAS-dcr2; R14H06-Gal4 flies were crossed to* different long hairpin *UAS-RNAi* lines (Valium 10) in the AttP2 landing site. *UAS-dcr2* was used to improve efficiency of the long hairpin RNAi. The AttP2 genetic background strain (BDSC36303) was crossed to *UAS-dcr2; R14H06-Gal4* as a control. (C) Same as in panel-B, but the *UAS-RNAi* lines were in the AttP40 landing site and the appropriate genetic background control line was used (BDSC36304). (D) *Tub-Gal80^{ts}; R14H06-Gal4* flies were crossed to *UAS-RNAi* lines from the TRiP collection in the Attp40 genetic background, and the appropriate genetic background control (BDSC36304). The experimental flies were maintained at 25ºC during development and the temperature was raised up to 29ºC 24 hours before the experiment. (E) Same as Panel D, but the RNAi lines were from the VDRC KK collection, and the appropriate genetic background control was used (VDRC60100). (F) *Tub-Gal80^{ts}; R14H06-Gal4* flies were crossed to *UAS-RNAi* lines from the TRiP collection in the AttP40 background. The experimental flies were maintained at 18ºC during development to avoid lethality and the temperature was raised to 29ºC 24 hours before the experiment. The appropriate genetic background control (BDSC36304) was used. (G, H) Same as in panel F, but the RNAi lines were TRiP *Attp2* insertions with the BDSC36303 control (G), or VDRC KK insertions with the VDRC60100 control (H). (I) Box plots showing courtship indices (CIs) for naive (N) and trained (T) flies with MB specific knockdown of *Hr38* and *sr*, and the genetic background control. AttP2 insertions from the TRiP collection and the genetic background control strain BDSC36303, were crossed to *Tub-Gal80^{ts}; R14H06-Gal4* and the progeny were raised at 18°C and transferred to 29°C 48 hours before STM training. (J) Box plot showing courtship indices (CIs) for naive (N) and trained (T) flies of UAS-only controls for *UAS-Hr38-RNAi* and *UAS-sr-RNAi* in LTM assays. RNAi lines were crossed to Nijmegen wild type flies to obtain the heterozygous *UAS-RNAi* flies. Statistical significance between naive and trained flies was determined using a two-tailed Mann-Whitney test. Mean is displayed with error bars indicating SEM. Number of flies tested for each condition is shown under the corresponding box plot. Source data are provided as a Source Data file

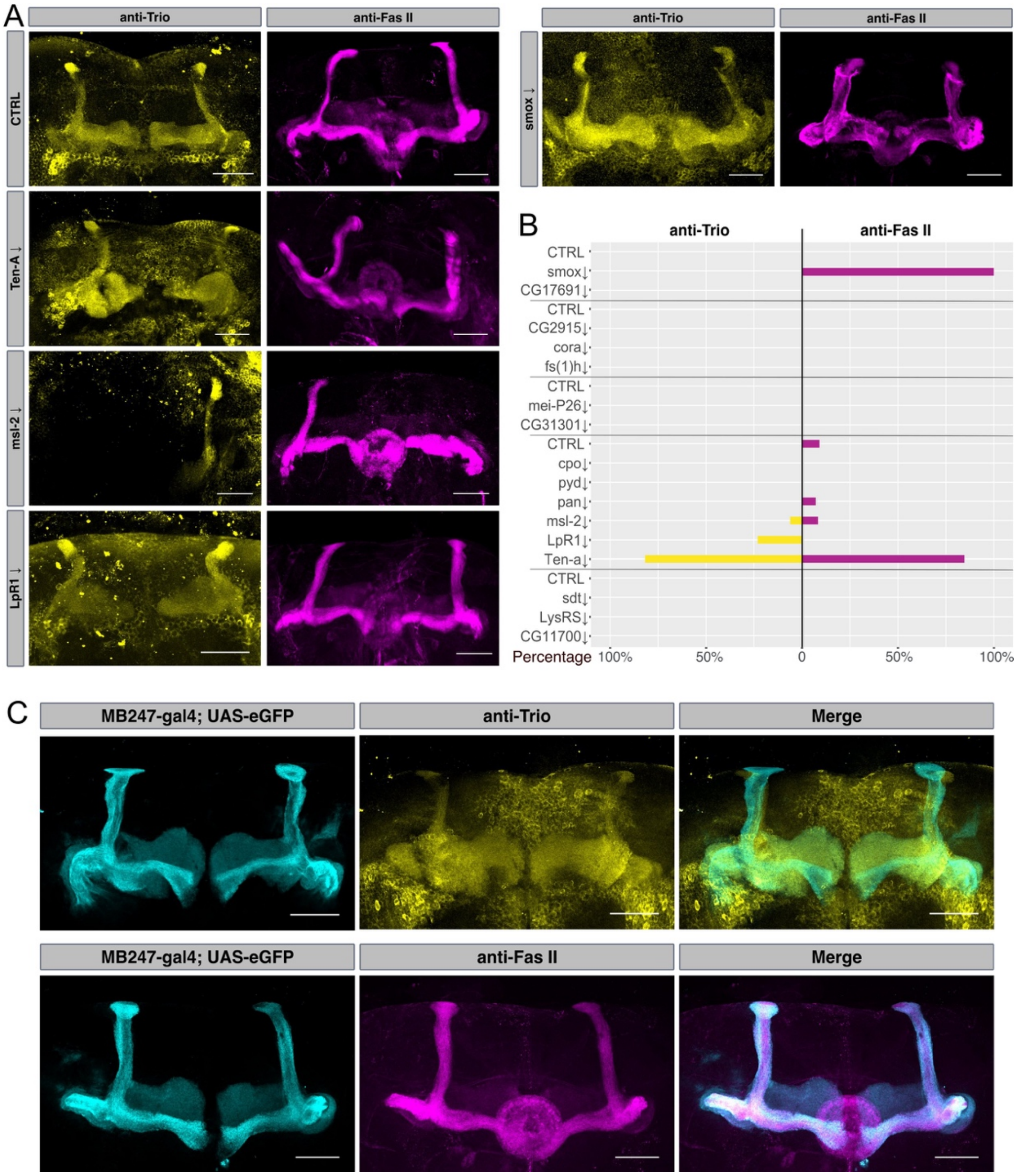

Figure S9 – Identification of MB developmental defects

(A) MB staining of positive hits with anti-TRIO (yellow) and fascicline II (magenta), which distinguish α´β´ and γ and αβ and γ lobes, respectively. Only a representative control and a representative altered MB are shown. Scale bar represents 50 μm. N>9. Source data are provided as a Source Data file. (B) Percentage of MB with alterations in the α´β´ or -γ lobes (yellow) and αβ or γ (magenta) respect to normal MB. For the Trio staining we only obtained significant p-values for Ten-A (0.0134). For FasII staining we obtained significant p-values for Ten-A (0.0307) and smox (0.0058). (C) Double staining of *MB247-gal4, UAS-eGFP* (cyan) with anti-TRIO (yellow) and fascicline II (magenta).

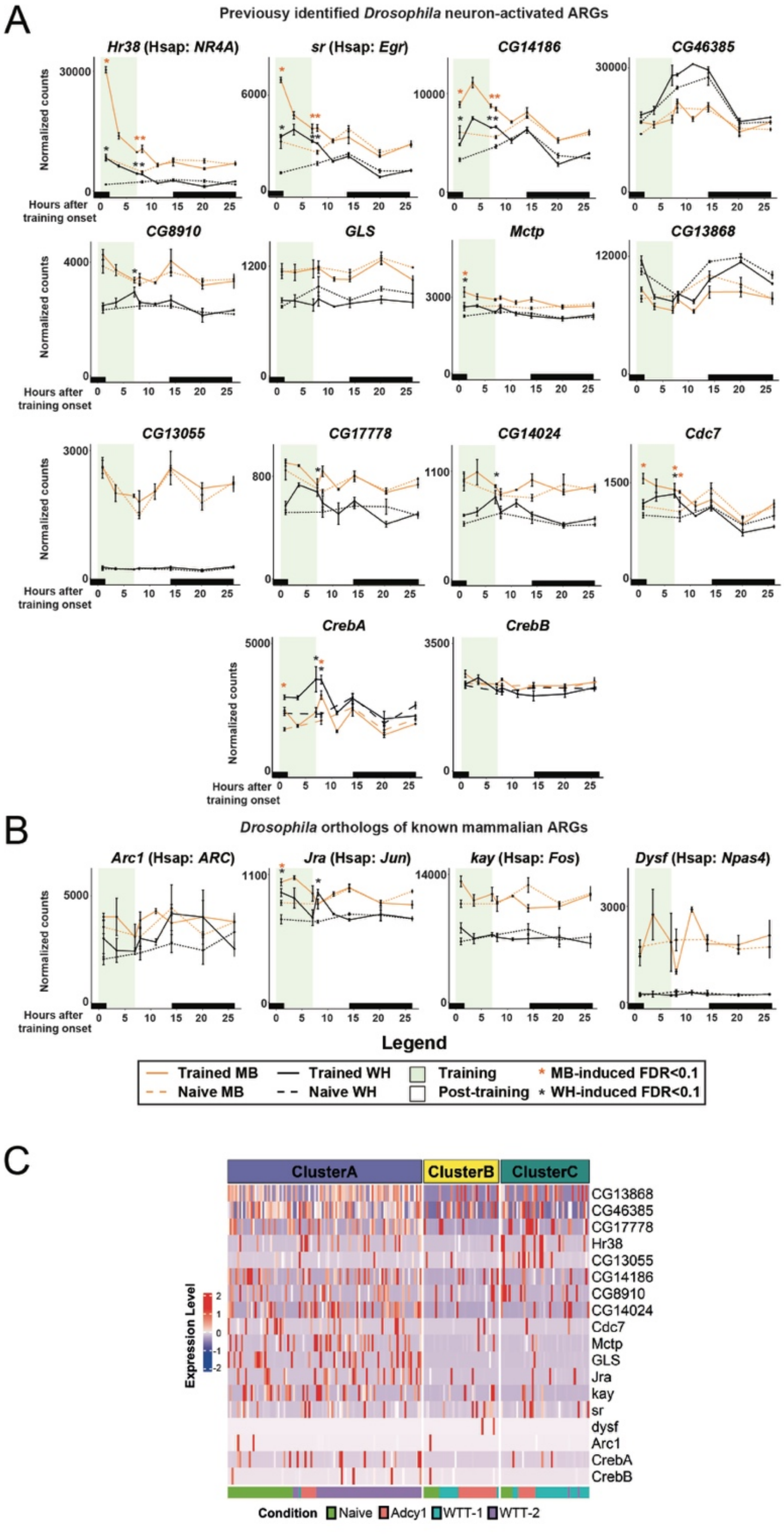
A
Previously identified Drosophila neuron-activated ARGs
Hr38 (Hsap: NR4A)
sr (Hsap: Egr)
CG14186
CG46385
CG8910
GLS
Mctp
CG13868
CG13055
CG17778
CG14024
Cdc7
CrebA
CrebB
Normalized counts
Hours after training onset
B
Drosophila orthologs of known mammalian ARGs
Arc1 (Hsap: ARC)
Jra (Hsap: Jun)
kay (Hsap: Fos)
Dysf (Hsap: Npas4)
Legend
Trained MB
Naive MB
Trained WH
Naive WH
Training
Post-training
MB-induced FDR<0.1
WH-induced FDR<0.1
C
ClusterA
ClusterB
ClusterC
Expression Level
CG13868
CG46385
CG17778
Hr38
CG13055
CG14186
CG8910
CG14024
Cdc7
Mctp
GLS
Jra
kay
sr
dysf
Arc1
CrebA
CrebB
Condition
Naive
Adcy1
WTT-1
WTT-2

Figure S10 – Candidate activity-regulated genes in courtship LTM. Normalized transcript levels of (A) 14 *Drosophila* activity-regulated genes (ARGs) previously identified[55] and (B) fly orthologs of known mammalian ARGs. Significance identified from differential expression analysis between trained and time-of-day matched naive flies, at time-points noted on the x-axis, is displayed (DESeq2, Wald's test, *FDR<0.1). Bold black line indicates incubator dark period. (C) Heatmap of the 14 *Drosophila* ARGs and for fly orthologs of known mammalian ARGs previously identified among the three ABC clusters. The lower bar in the graph indicates the experimental condition for each sequenced neuron.

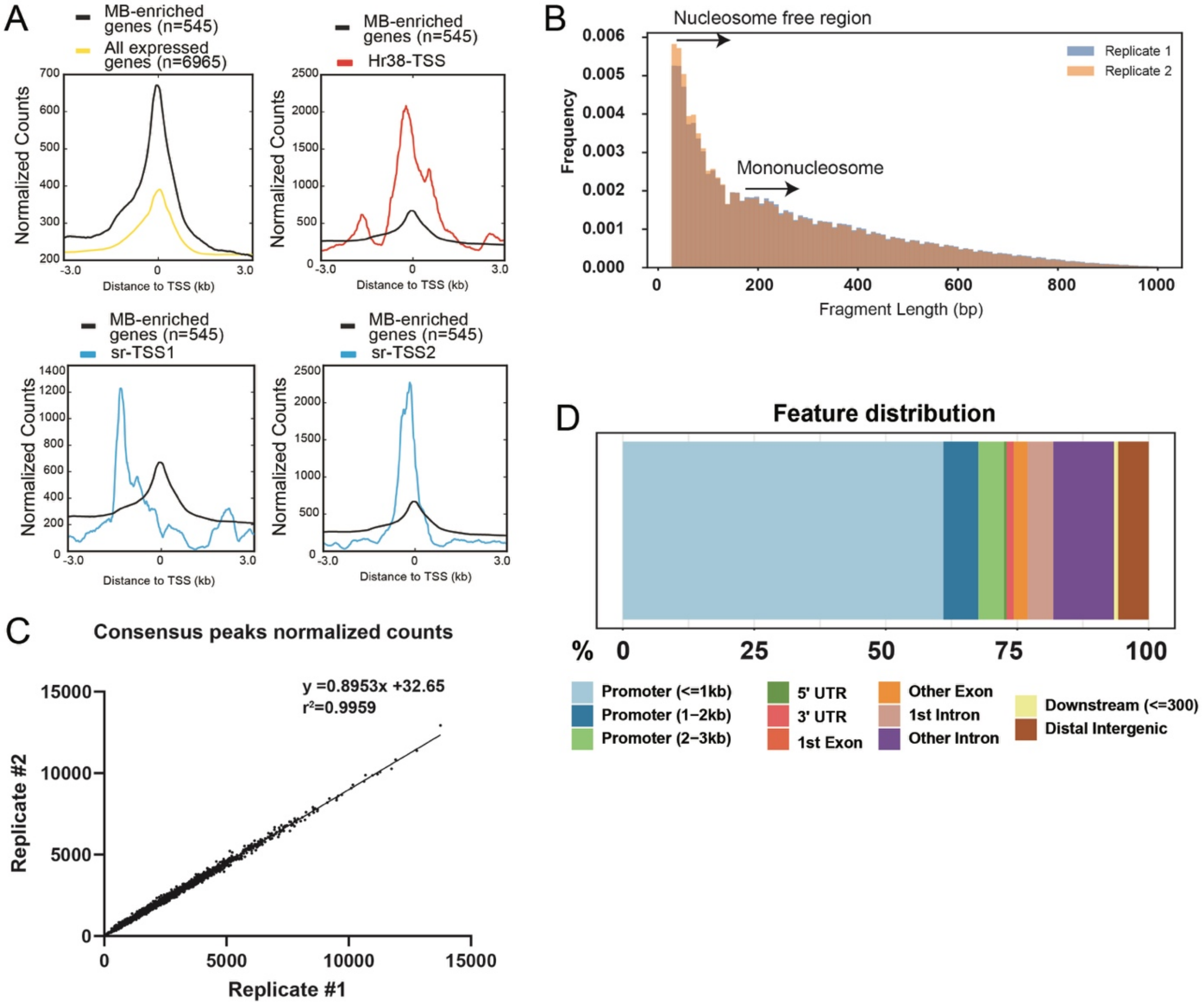

Figure S11 – ATAC-seq Quality Control. (A) Band plot of chromatin accessibility profiles for genes with enriched expression in the MB (n=545, see **Fig. S1B**) compared to all expressed genes (n=6965), and the transcriptional start sites (TSS) of *Hr38* and *sr*. (B) Fragment size distribution of ATAC-seq libraries generated from INTACT-isolated MB nuclei. The distribution for two biological replicates is shown. Peak signal of nucleosome free regions (80-120 bp) and mononucleosomes (~180 bp) is marked. (C) Scatter plot of normalized counts for consensus peaks from MB ATAC-seq samples. Each dot represents a consensus peak, with positions indicating normalized counts in replicate 1 and replicate 2. Line of best fit is shown, with corresponding linear equation, and coefficient of determination (R^2). (D) Feature distribution of annotated regulatory regions from significantly accessible peaks and is predominantly located near to the transcriptional start site (TSS) of genes.

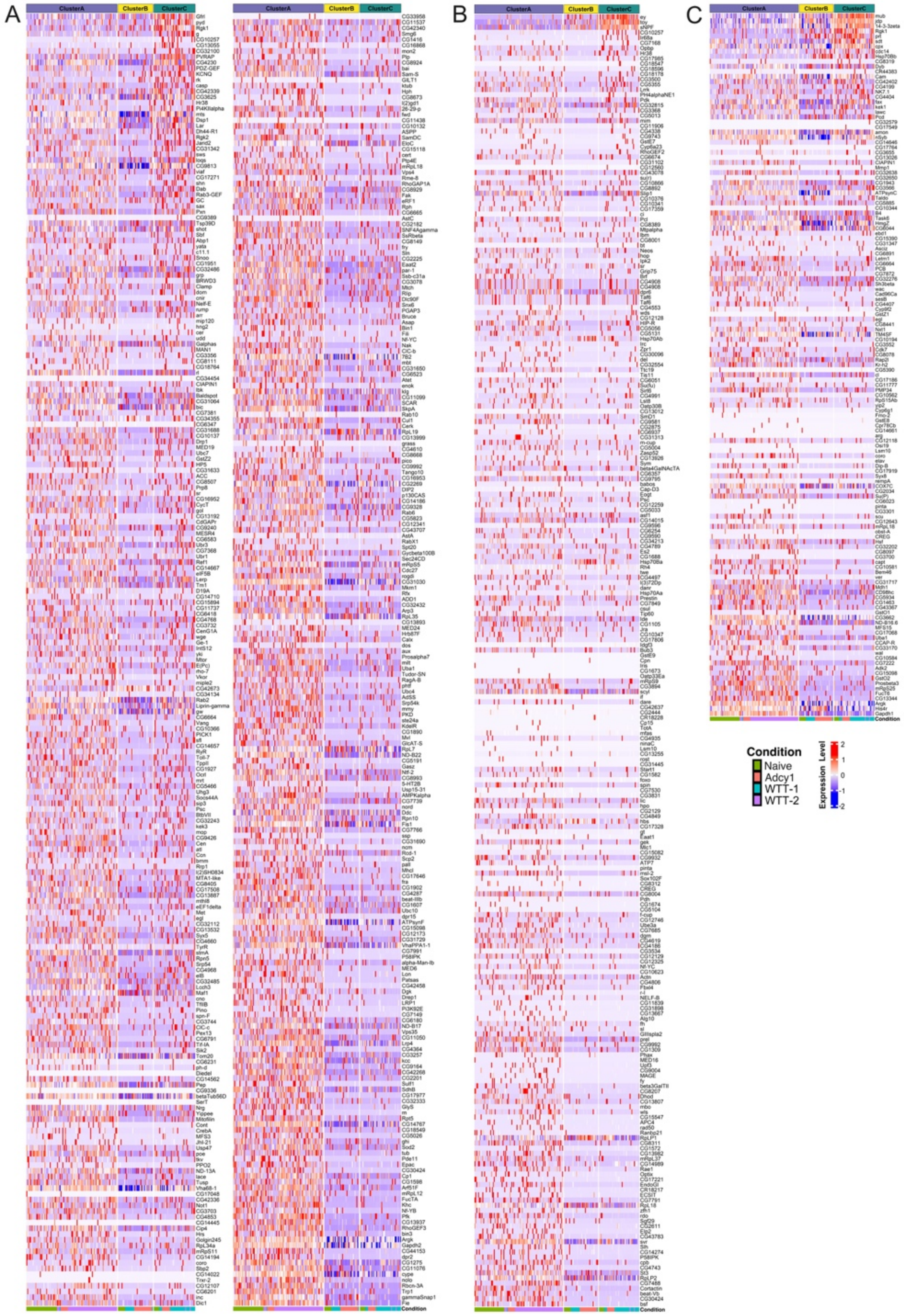

Figure S12 - Comparison of scRNAseq data with other datasets. (A) Heatmap of up-regulated genes (Fcross<0.05, N=635) of dopaminergic neurons after group housing (from

Agrawal et al., 2019) (B) Heatmap of up-regulated genes ($p<0.1$, N=366) from DAL, MBONa3, γ KCs and V2 neurons after olfactory aversive conditioning (from Crocker et al, 2016). (C) Heatmap of genes with increased RNApol II occupancy in the MB (N=288) by Targeted DamID after olfactory appetitive conditioning (from Widmer et al., 2018). In all lists genes are ordered by their expression levels in cluster C

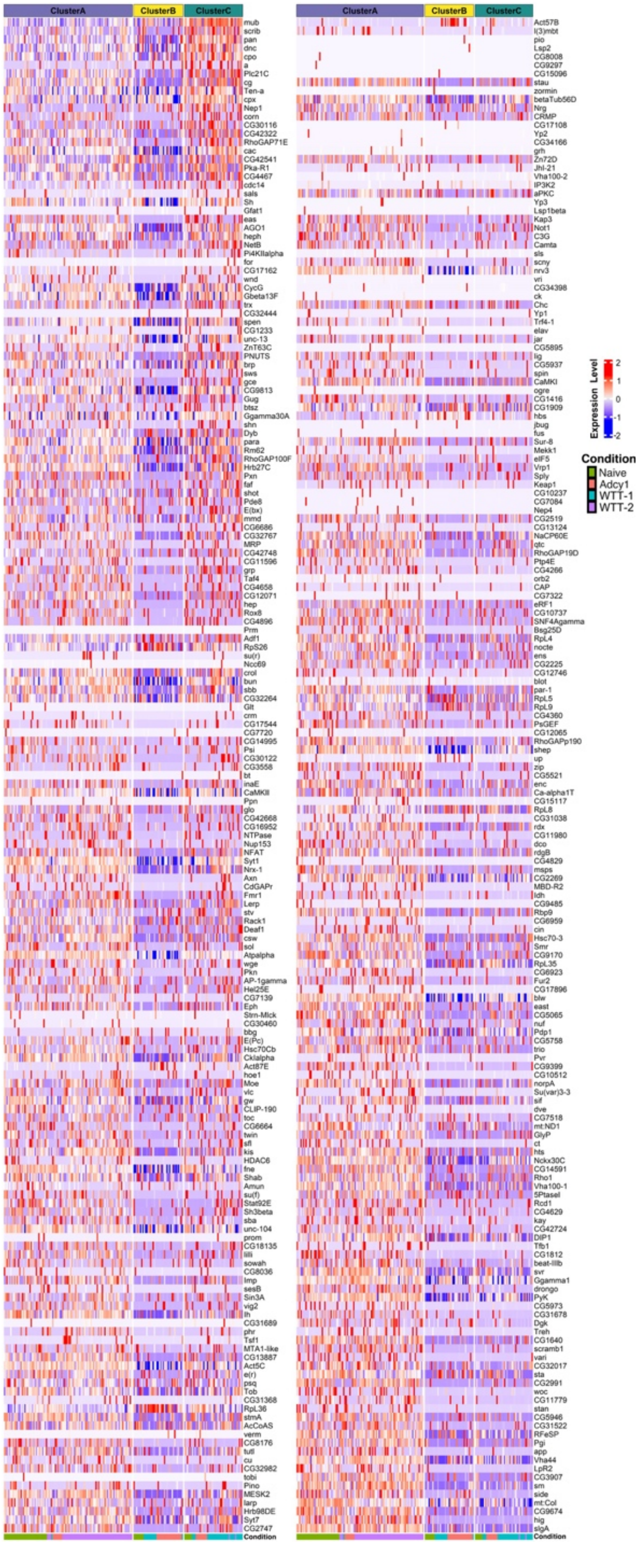

Figure S13 - Comparison of scRNAseq data with dataset from Winbush et al. Heatmaps of candidate up-regulated genes (N=480) of whole heads after courtship conditioning (from Winbush et al., 2012). Genes are ordered by their expression levels in cluster C
