# Supplementary material for: A memory transcriptome time course reveals essential long-term memory transcription factors
Source: Nat Commun. 2025 Oct 29;16:9320. doi: 10.1038/s41467-025-64379-x (PMC12572301; doi:10.1038/s41467-025-64379-x)
Supplement: Supplementary file 2 — Description of Additional Supplementary Files [file 41467_2025_64379_MOESM2_ESM.pdf]

Description of Additional Supplementary Files

Supplementary Data 1: Differentially expressed genes in whole head (naive vs trained)

Supplementary Data 2: Differentially expressed genes in mushroom body (naive vs trained)

Supplementary Data 3: GO enrichment for training induced genes

Supplementary Data 4: Overlap of courtship memory transcriptome datasets using MB INTACT (1 hour after the end of training)

Supplementary Data 5: GO enrichment of scRNAseq clusters 1-5 and normalized counts

Supplementary Data 6: Mushroom body enriched genes (mushroom body vs whole head)

Supplementary Data 7: GO enrichment of scRNAseq clusters ABC

Supplementary Data 8: List of selected candidate genes for courtship conditioning LTM screen

Supplementary Data 9: Crosses and genotypes

Supplementary Data 10: MB-specific ATAC-seq peak list

Supplementary Data 11: Training induced genes and top expressed genes in cluster ABC bound by Hr38, sr, and CREBB

Supplementary Data 12: Overlap of training induced genes with datasets using different tissues and memory paradigms
