# Supplementary material for: A memory transcriptome time course reveals essential long-term memory transcription factors
Source: Nat Commun. 2025 Oct 29;16:9320. doi: 10.1038/s41467-025-64379-x (PMC12572301; doi:10.1038/s41467-025-64379-x)
Supplement: Supplementary file 15 — Reporting Summary [file 41467_2025_64379_MOESM15_ESM.pdf]

nature portfolio

Corresponding author(s): Francisco A Martin
Jamie M Kramer

Last updated by author(s): Jun 1, 2025

Reporting Summary

Nature Portfolio wishes to improve the reproducibility of the work that we publish. This form provides structure for consistency and transparency in reporting. For further information on Nature Portfolio policies, see our Editorial Policies and the Editorial Policy Checklist.

Statistics

For all statistical analyses, confirm that the following items are present in the figure legend, table legend, main text, or Methods section.

n/a	Confirmed	
☐	☒	The exact sample size (*n*) for each experimental group/condition, given as a discrete number and unit of measurement
☐	☒	A statement on whether measurements were taken from distinct samples or whether the same sample was measured repeatedly
☐	☒	The statistical test(s) used AND whether they are one- or two-sided<br>*Only common tests should be described solely by name; describe more complex techniques in the Methods section.*
☐	☒	A description of all covariates tested
☐	☒	A description of any assumptions or corrections, such as tests of normality and adjustment for multiple comparisons
☐	☒	A full description of the statistical parameters including central tendency (e.g. means) or other basic estimates (e.g. regression coefficient) AND variation (e.g. standard deviation) or associated estimates of uncertainty (e.g. confidence intervals)
☒	☐	For null hypothesis testing, the test statistic (e.g. *F*, *t*, *r*) with confidence intervals, effect sizes, degrees of freedom and *P* value noted<br>*Give P values as exact values whenever suitable.*
☒	☐	For Bayesian analysis, information on the choice of priors and Markov chain Monte Carlo settings
☒	☐	For hierarchical and complex designs, identification of the appropriate level for tests and full reporting of outcomes
☒	☐	Estimates of effect sizes (e.g. Cohen's *d*, Pearson's *r*), indicating how they were calculated

Our web collection on statistics for biologists contains articles on many of the points above.

Software and code

Policy information about availability of computer code

Data collection	For bulk RNAseq and ATAC-seq: first, raw reads were lightly trimmed, and adaptors clipped using Trimmomatic (v0.39). The read quality was assessed using FastQC (v0.11.9) and trimmed reads were aligned to the Drosophila melanogaster genome (Ensembl release 103, dm6) using STAR (v2.7.5a) and bowite2 (v2.4.1) for RNAseq and ATAC-seq data, respectively. For RNAseq, uniquely aligned reads with a maximum of four mismatches were counted to genes using featureCounts. For ATACseq data, reads aligning to multiple loci, the mitochondrial genome, and scaffolds were filtered using samtools view (v1.11); peaks were called using MACS2 software (v2.1.2); consensus peaks were annotated using the R Package ChIPseeker (v1.26.2); DiffBind (v3.0.15) was used to determine the fraction of reads in peaks calculated (FRiP).<br>For scRNAseq: FASTQ reads were quality checked using FastQC1 (v0.11.9) software and aligned against the Drosophila melanogaster reference genome release 6 (Ensembl release 103, dm6) with STAR (v2.7.9) aligner. Htseq-count (v0.11.2) was then used to count the reads mapping each annotated feature
Data analysis	For bulk RNAseq: differential expression analysis was done using DESeq2 (v1.30.1)74 in Rstudio (v4.0.3). Gene annotation, gene ontology (GO) and statistical comparison between groups of genes were performed using the R package BinfTools (https://github.com/kevincjnixon/BinfTools). Data was further visualized using the R packages ggplot2 (v3.4.2) and pheatmap (v1.0.12). Venn diagrams were created using BioVenn.<br>For ATACseq: For visualization of ATAC data, promoter and genomic regions were extracted using the R annotation package TxDb.Dmelanogaster.UCSC.dm6.ensGene (v3.12.0) in combination with GenomicRanges (v1.42.0)<br>For ChIP-seq: publicly available ChIP-seq data was obtained from the ENCODE project repository. ChIP-seq peaks for Hr38, Sr, and CrebB, were generated using optimal IDR thresholding by ENCODE. These peaks were annotated to the nearest gene using ChIPseeker (v1.26.2). Genome browser tracks were generated with pyGenomeTracks.<br>For scRNAseq: The obtained gene expression matrix was used as input to perform downstream analyses in Seurat v4.03. Differential marker

gene lists were represented through heatmap plots using ComplexHeatmap R package. Differentially expressed genes from each single cell cluster were analyzed for GO and KEGG term enrichment using the R package ClusterProfiler

For manuscripts utilizing custom algorithms or software that are central to the research but not yet described in published literature, software must be made available to editors and reviewers. We strongly encourage code deposition in a community repository (e.g. GitHub). See the Nature Portfolio guidelines for submitting code & software for further information.

Data

Policy information about availability of data

All manuscripts must include a data availability statement. This statement should provide the following information, where applicable:

- Accession codes, unique identifiers, or web links for publicly available datasets
- A description of any restrictions on data availability
- For clinical datasets or third party data, please ensure that the statement adheres to our policy

Raw and processed RNA-seq and ATAC-seq data are submitted to the GEO database under accession numbers GSE282414 and GSE274348. All raw single cell RNAseq data were uploaded to European Nucleotide Archive (ENA) under the Accession number PRJEB49180.

Research involving human participants, their data, or biological material

Policy information about studies with human participants or human data. See also policy information about sex, gender (identity/presentation), and sexual orientation and race, ethnicity and racism.

Reporting on sex and gender	*Use the terms sex (biological attribute) and gender (shaped by social and cultural circumstances) carefully in order to avoid confusing both terms. Indicate if findings apply to only one sex or gender; describe whether sex and gender were considered in study design; whether sex and/or gender was determined based on self-reporting or assigned and methods used. Provide in the source data disaggregated sex and gender data, where this information has been collected, and if consent has been obtained for sharing of individual-level data; provide overall numbers in this Reporting Summary. Please state if this information has not been collected. Report sex- and gender-based analyses where performed, justify reasons for lack of sex- and gender-based analysis.*
Reporting on race, ethnicity, or other socially relevant groupings	*Please specify the socially constructed or socially relevant categorization variable(s) used in your manuscript and explain why they were used. Please note that such variables should not be used as proxies for other socially constructed/relevant variables (for example, race or ethnicity should not be used as a proxy for socioeconomic status). Provide clear definitions of the relevant terms used, how they were provided (by the participants/respondents, the researchers, or third parties), and the method(s) used to classify people into the different categories (e.g. self-report, census or administrative data, social media data, etc.) Please provide details about how you controlled for confounding variables in your analyses.*
Population characteristics	*Describe the covariate-relevant population characteristics of the human research participants (e.g. age, genotypic information, past and current diagnosis and treatment categories). If you filled out the behavioural & social sciences study design questions and have nothing to add here, write "See above."*
Recruitment	*Describe how participants were recruited. Outline any potential self-selection bias or other biases that may be present and how these are likely to impact results.*
Ethics oversight	*Identify the organization(s) that approved the study protocol.*

Note that full information on the approval of the study protocol must also be provided in the manuscript.

Field-specific reporting

Please select the one below that is the best fit for your research. If you are not sure, read the appropriate sections before making your selection.

☒ Life sciences ☐ Behavioural & social sciences ☐ Ecological, evolutionary & environmental sciences

For a reference copy of the document with all sections, see nature.com/documents/nr-reporting-summary-flat.pdf

Life sciences study design

All studies must disclose on these points even when the disclosure is negative.

Sample size	The initial sample size for each condition in Courtship Conditioning experiments (Fig 3C, 3E, 7C, 7E, Fig S8) was at least 15 flies. For bulk RNAseq experiments, 40 to 50 fly heads were pooled per replicate, with two separate pools as biological replicates, as described previously (.Jones et al, G3 2018). For scRNAseq experiments, 40 to 50 dissected brains were dissociated per condition. The size of CAMEL-GFP sample (Fig 4B) was at least 11 hemispheres per condition. The number of stained MB per condition (Fig S9) was 10-19 (two replicates). The RT-PCR (fig 8C-D)was performed used 20 flies per replicate
Data exclusions	In Courtship Conditioning experiments, the replicate was discarded when the correspondant control sample did not render significant differences between trained and naive flies. We did not rule out any data from CAMEL-GFP experiment.

Replication	For Courtship Conditioning experiments, each condition was repeated between 2-4 times. Given the similarity of collected data, we combined all the non-excluded replicates for the statistical analysis. Two biological replicates were obtained from the CAMEL-GFP experiment. For the RT-PCR, the number of replicates is 3.
Randomization	All flies from the same genotype were collected together and randomly selected to be trained or kept as a naive control
Blinding	To avoid bias, in the Courtship Conditioning experiment the analysis for each condition was performed blindly, using a two letter-number code. Naive/trained condition was known by the researcher.

Reporting for specific materials, systems and methods

We require information from authors about some types of materials, experimental systems and methods used in many studies. Here, indicate whether each material, system or method listed is relevant to your study. If you are not sure if a list item applies to your research, read the appropriate section before selecting a response.

Materials & experimental systems

n/a	Involved in the study	
☐	☒	Antibodies
☒	☐	Eukaryotic cell lines
☒	☐	Palaeontology and archaeology
☐	☒	Animals and other organisms
☒	☐	Clinical data
☒	☐	Dual use research of concern
☒	☐	Plants

Methods

n/a	Involved in the study	
☒	☐	ChIP-seq
☐	☒	Flow cytometry
☒	☐	MRI-based neuroimaging

Antibodies

Antibodies used	rabbit anti-GFP (1/200, Invitrogen ref. A11122), mouse anti-Fasciclin II (1/25; DSHB AB_528235), anti-Trio (1/25; DSHB AB_528494), anti-GFP (1/300; Invitrogen G10362), anti-Dac (1/50; DSHB mAbdac1-1), Alexia 488 and 568 (1/500 or 1/300, respectively; Life Technologies).
Validation	By confocal microscopy, with the same expression pattern as previously described. Also, other labs have validated all used antibodies

Animals and other research organisms

Policy information about studies involving animals; ARRIVE guidelines recommended for reporting animal research, and Sex and Gender in Research

Laboratory animals	DRosophila melanogaster
Wild animals	The study did not involve wild animals
Reporting on sex	Findings only applied to one sex, given that the learning assay employed (Courtship conditioning) uses courtship, a behavior that is exclusive of males. We assigned sex by the abdomen morphology and behavior during courtship
Field-collected samples	The study did not involve samples collected in the field
Ethics oversight	No ethical aproval or guidance was required for Drosophila melanogaster

Note that full information on the approval of the study protocol must also be provided in the manuscript.

Plants

Seed stocks	*Report on the source of all seed stocks or other plant material used. If applicable, state the seed stock centre and catalogue number. If plant specimens were collected from the field, describe the collection location, date and sampling procedures.*
Novel plant genotypes	*Describe the methods by which all novel plant genotypes were produced. This includes those generated by transgenic approaches, gene editing, chemical/radiation-based mutagenesis and hybridization. For transgenic lines, describe the transformation method, the number of independent lines analyzed and the generation upon which experiments were performed. For gene-edited lines, describe the editor used, the endogenous sequence targeted for editing, the targeting guide RNA sequence (if applicable) and how the editor was applied.*
Authentication	*Describe any authentication procedures for each seed stock used or novel genotype generated. Describe any experiments used to assess the effect of a mutation and, where applicable, how potential secondary effects (e.g. second site T-DNA insertions, mosiacism, off-target gene editing) were examined.*

Flow Cytometry

Plots

Confirm that:

☒ The axis labels state the marker and fluorochrome used (e.g. CD4-FITC).

☒ The axis scales are clearly visible. Include numbers along axes only for bottom left plot of group (a 'group' is an analysis of identical markers).

☒ All plots are contour plots with outliers or pseudocolor plots.

☒ A numerical value for number of cells or percentage (with statistics) is provided.

Methodology

Sample preparation	40 to 50 brain were dissected removing optical lobes and trachea, dissociated following Davie et al (Cell, 2018) and sorted straight into a 96-well plate with the lysis buffer to carry out single cell genomics
Instrument	Influx cell sorter (Becton Dickinson) equipped with a 355nm and 488nm laser lines
Software	BD FACSTM Sortware v1.0
Cell population abundance	We selected GFP positive cells: for naive samples, 0.12%; for trained 0.24%; for rut-trained, 0.08%
Gating strategy	We exclude aggregates using pulse processing and dead cells using DAPI as a viability dye. The gating strategy is shown in fig S1

☒ Tick this box to confirm that a figure exemplifying the gating strategy is provided in the Supplementary Information.
